# Supplementary material for: Microfilaremic loiasis is associated with T cell hyporesponsiveness against SARS-CoV-2
Source: PLoS Negl Trop Dis. 2026 Oct 1;20(10):e0014104. doi: 10.1371/journal.pntd.0014104 (PMC13652322; doi:10.1371/journal.pntd.0014104)

S4: Spearman's rank correlation showing weak correlations of absolute IFN-γ and absolute eosinophil numbers (A) as well as correlation of absolute IFN-γ and microfilaremia (B).

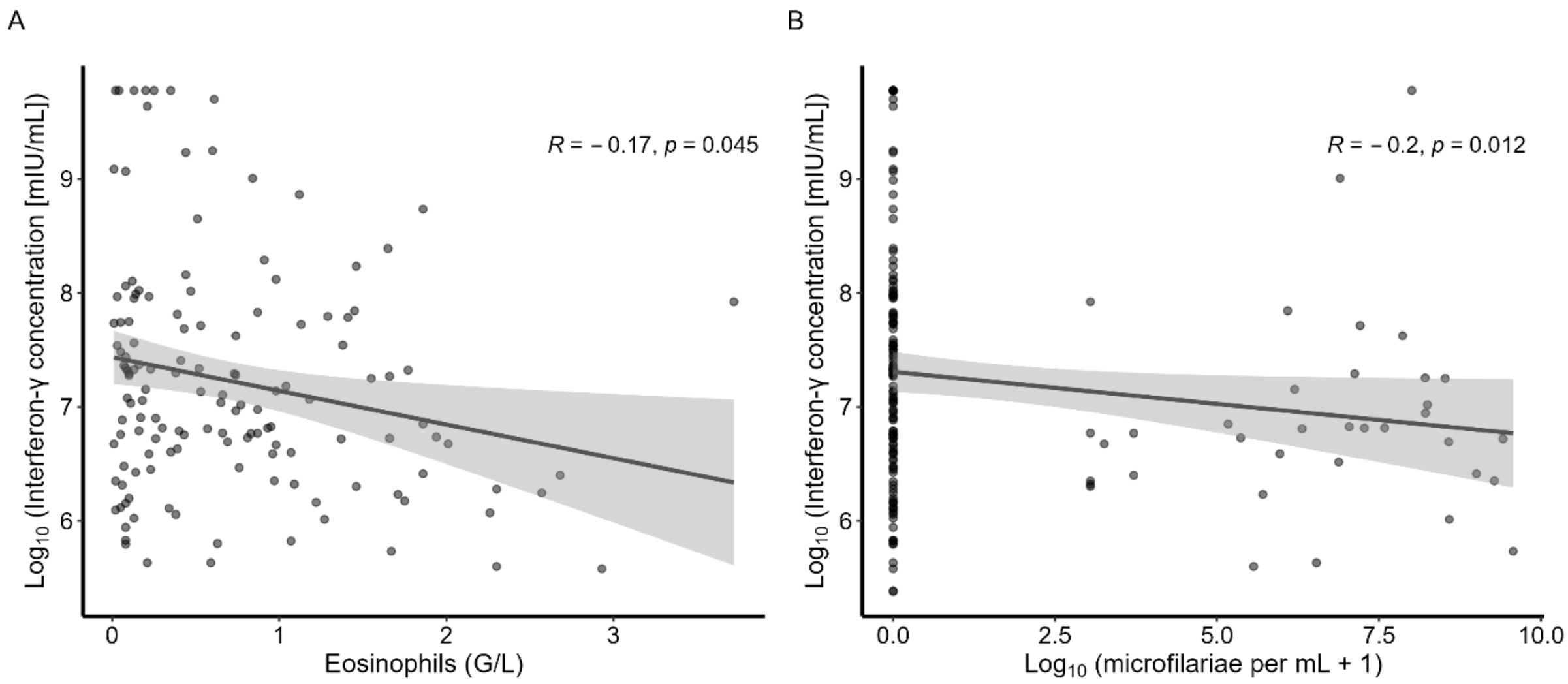

Supplement: Supplementary file 1 [file pntd.0014104.s007.pdf]
