# Supplementary material for: Microfilaremic loiasis is associated with T cell hyporesponsiveness against SARS-CoV-2
Source: PLoS Negl Trop Dis. 2026 Oct 1;20(10):e0014104. doi: 10.1371/journal.pntd.0014104 (PMC13652322; doi:10.1371/journal.pntd.0014104)
Supplement: Supplementary file 2 [file pntd.0014104.s009.docx]

Response to reviewers PNTD-D-26-00408
Microfilaremic loiasis is associated with T cell hyporesponsiveness against SARS-CoV-2
PLOS Neglected Tropical Diseases

Dear Editors,

we are very grateful for your consideration of our manuscript “Microfilaremic loiasis is associated with T cell hyporesponsiveness against SARS-CoV-2”. We have addressed all reviewers` comments and hope that our manuscript will now be deemed suitable for publication by PlosNTD.

Please find our point-by-point responses below.

On behalf of all the coauthors,

Yours sincerely,

Luzia Veletzky, Rafiou Adamou, Maria Mackroth

Hamburg, Lambaréné, Vienna

09^th^ June 2026


**Methods**
-Are the objectives of the study clearly articulated with a clear testable hypothesis stated?
-Is the study design appropriate to address the stated objectives?
-Is the population clearly described and appropriate for the hypothesis being tested?
-Is the sample size sufficient to ensure adequate power to address the hypothesis being tested?
-Were correct statistical analysis used to support conclusions?
-Are there concerns about ethical or regulatory requirements being met?
Reviewer #1: See general comments

Reviewer #2: Yes to all. Very well designed study.

Reviewer #3: (No Response)

**Results**
-Does the analysis presented match the analysis plan?
-Are the results clearly and completely presented?
-Are the figures (Tables, Images) of sufficient quality for clarity?

Reviewer #1: See general comments

Reviewer #2: Yes. See notes below -- would likely benefit for inclusion of the results of univariate and multivariate modeling for all the possible confounders evaluated.

**ANSWER:** Please find our answers below.

Reviewer #3: Suggestions:

- Differences of eosinophils and lymphocytes were normalized based on sex and age. What happens with these parameters once you stratify the groups according to the presence of co-infections?

- Additional analysis, for IgG and IFN, should be done stratifying the groups regarding the presence of co-infections. Each group should be divided into no-coinfection and any co-infection.

-Specifically for the IFN results it would be interesting to stratify the groups not necessarily regarding all the co-infections but separating the individuals that present co-infections that are known to induce type I immune responses.

-Besides the eosinophil, is there any correlation regarding the lymphocytes and the IFN?

**ANSWER:** Please find our answers below.

**Conclusions**
-Are the conclusions supported by the data presented?
-Are the limitations of analysis clearly described?
-Do the authors discuss how these data can be helpful to advance our understanding of the topic under study?
-Is public health relevance addressed?

Reviewer #1: See general comments

Reviewer #2: Yes.

Reviewer #3: (No Response)

**Editorial and Data Presentation Modifications?**
Use this section for editorial suggestions as well as relatively minor modifications of existing data that would enhance clarity. If the only modifications needed are minor and/or editorial, you may wish to recommend "Minor Revision" or "Accept".

Reviewer #1: See general comments

Reviewer #2: (No Response)

Reviewer #3: (No Response)

**Summary and General Comments**

**Reviewer #1:**

**COMMENT:** This study addresses an important and understudied question regarding how chronic helminth infections such as Loa loa may influence immune responses to unrelated pathogens. Understanding such interactions is particularly relevant in endemic settings where helminth infections remain common and may influence responses to other infectious pathogens or vaccination.
The abstract states that the study was done in "Lambaréné and surrounding rural areas of Gabon" while the methods (lines 137-138) states "rural villages of the Ngounié and Moyen-Ogooué provinces of Gabon". Lambarene presumably includes urban populations sop this contradiction needs to be clarified.

**ANSWER**: Dear reviewer, thank you for pointing this out. We have clarified this part in the text. Even though Lambaréné is considered a city, most of the population will at least spend some time on the countryside and often have a “rural” lifestyle. Participants were included in the periruban regions of Lambaréné and rural villages.

**CHANGES** Line 54-55: Between 2022 and 2024 this cross-sectional study was done in rural and periurban villages of the Ngounié and Moyen-Ogooué provinces of Gabon.

Line 142-143: This cross-sectional study was conducted between 2022 and 2024 in rural and periurban villages of the Ngounié and Moyen-Ogooué provinces of Gabon.


**COMMENT:** RAPLOA was developed as a population-level mapping tool rather than a diagnostic instrument for individual classification. Because it relies on recall of of eyeworm migration, it may not reliably reflect current infection status. The resulting "occult loiasis" category likely will include biologically heterogeneous individuals, including those whose immune systems control microfilaremia more effectively and who mount stronger anti-parasite immune responses. Such individuals might also mount stronger IFN-γ responses to unrelated antigens, including SARS-CoV-2. Sensitivity analyses could help address this issue, including (i) comparing microfilaremia versus no microfilaremia independent of RAPLOA stratification and (ii) examining correlations between microfilarial load and IFN-γ responses among microfilaremic individuals.

**ANSWER:** Dear reviewer, we fully agree that these are important points. The issue of how to diagnose or especially exclude an “active occult infection” remains challenging. To account for this, we did first assess the differences between microfilaremic vs non-microfilaremic (NM, including all other than microfilaremic participants) and this is shown in Fig 2 upper panel and described in lines 296-313.

For the second point (ii) raised, we present the results in Lines 340-344: We next examined correlations between IFN-γ concentrations and parasitological or hematological parameters. Absolute IFN-γ concentrations and absolute eosinophil numbers showed a weak negative correlation (rho = -0.17, p=0.045) as did absolute IFN-γ and microfilaremia (rho=-0.2, p=0.012), as tested using spearman’s rank correlation coefficient (Supp. Info. S6).

To further underline these issues we have added a paragraph in the discussion:

**CHANGES** Line 427-434: In line with this argument one may consider that the lack of difference between NL and OL, may also be due to the fact that diagnosing active occult infection in absence of eyeworm migration or excluding an active occult infection is currently nearly impossible by available methods. Thus, individuals of the “occult loiasis” and “no sign of infection group” may have been misclassified which consequently could have concealed differences between true occult and true negatives. Individuals whose immune system is efficient to control microfilaremia and/or primary infection may mount stronger IFN-γ responses to unrelated antigens, including SARS-CoV-2.

**COMMENT:** Could the authors provide a clearer explanation for the following: The relative validation criteria for the IFN-γ release assay led to some Quan-T-Cell-test-negative individuals having a higher measured IFN-γ concentration than some positive individuals".

A potentially important issue relates to the exclusion of IFN-g-'negative' individuals from analysis. Because IFN-γ responsiveness is the outcome of interest, restricting the analysis to individuals with detectable responses means selecting participants based on the outcome itself, which may introduce bias. An alternative approach could be to analyse all participants and model either responder status or IFN-γ concentrations including zero values or to model response probability (responder vs. non-responders).

**ANSWER:** Dear reviewer, we completely understand your comment. We agree that excluding IFN-γ non-responders could potentially introduce bias. We have repeatedly discussed this issue in the group of immunologists and statisticians, to take the immunological, statistical and biological rationale into account.

To clarify, classification as a responder (Quan-T cell positive) or non-responder (Quan-T cell negative) was based on the manufacturer's validated interpretation criteria for the Quan-T-Cell SARS-CoV-2 assay rather than on the absolute IFN-γ concentration measured after antigen stimulation. Specifically, a sample is considered positive (responsive) only when the antigen-specific IFN-γ response exceeds 1.5 times the individual background (blank) value. Because background IFN-γ concentrations vary substantially between individuals, some participants classified as non-responders may nevertheless exhibit higher absolute net IFN-γ concentrations (Sars-CoV2 response minus blank) than participants classified as responders. Consequently, interpretation of antigen-specific responses in assay-negative individuals is challenging, as elevated IFN-γ values may reflect background variability rather than a confirmed SARS-CoV-2-specific response. Furthermore, some assay-negative individuals may never have been exposed to SARS-CoV-2, whereas others may have had prior exposure but no detectable cellular response at the time of testing.

For this reason, our primary analysis focused on individuals classified as responders according to the validated assay criteria. However, in response to the reviewer's concern, we performed an additional sensitivity analysis including all participants. In this model, net IFN-γ concentrations of all study participants with valid IFN-γ assay results were analyzed while adjusting for age, sex, and responder status (yes/no). The results are now presented in Supp. Info. S4. Importantly, this analysis showed the same overall pattern as the primary analysis. The differences between non-microfilaraemic and microfilaraemic as well as between occult loiasis and microfilaremic individuals remained consistent. These findings support the robustness of our conclusions and indicate that the observed associations are not driven by the exclusion of assay-negative participants. Of note, the percentage of non-responders did not vary between groups with 10 in group NL, 6 in group OL and 5 in group ML.

We have added a clarification of the assay interpretation criteria and included the additional sensitivity analysis in the revised manuscript:

**CHANGES** Line 183-191: A stimulation response was considered negative (non-responsive) if it did not exceed 150% of the blank value. Because Quan-T-Cell positivity is defined relative to the individual's background IFN-γ production rather than by an absolute IFN-γ concentration threshold, some assay-negative individuals exhibited higher net IFN-γ concentrations than some assay-positive individuals. Therefore, the primary analysis was restricted to individuals classified as responders according to the manufacturer's validated criteria. To assess the robustness of this approach, we additionally performed a sensitivity analysis including all participants (responder and non-responder) with valid assay results and adjusting for responder status.

**CHANGES** Line 291-331: Additional 6 participants (3.2%) were excluded from statistical analysis due to invalid results. 21 participants (11%) were considered non-responders to SARS-CoV-2 according to the manufacturer's validated criteria of the Quan-T-cell-Assay. Of the 21 non-responders, 5 belonged to the microfilaremic group, 6 to the occult loiasis group, and 10 to the group without signs of active infection. Thus, 162 Loa-exposed participants (84.4%) showed detectable IFN-γ responses to SARS-CoV-2 and were classified as responders.

[…]

The primary analyses of SARS-CoV-2-specific IFN-γ responses were restricted to responders according to the validated assay criteria.

[…]

To assess the robustness of these findings, an additional sensitivity analysis including all participants with valid assay results was performed, with multivariable models adjusting for age, sex, and responder status (Supp. Info. S4). The sensitivity analysis reproduced the pattern observed in the primary analysis, with a significant difference between microfilaraemic and non-microfilaraemic individuals persisting after adjustment for age, sex, and responder status (p = 0.031).

[…]

An additional sensitivity analysis of the IFN-γ responses was performed, including all participants with valid assay results (responder and non-responder). Multivariable models were adjusted for age, sex, and responder status. The association between microfilaremia and lower SARS-CoV-2-specific IFN-γ responses remained consistent, with microfilaremic individuals exhibiting significantly lower IFN-γ responses than participants with occult loiasis after adjustment for the covariates (p= 0.012) (Supp. Info. S4).

**COMMENT:** Details should be provided of the laboratory detection of STH/Schistosoma in stool and urine samples

**ANSWER:** We have added the details on the methods in Supporting information S1.

**CHANGES** Line 169-172: Additionally, thick blood smears were examined for detection of Plasmodium spp. Stool and urine samples were examined to identify soil-transmitted helminths, intestinal protozoa and Schistosoma eggs using established protocols [20], for detailed protocols please see Supporting Information S1.

**COMMENT:** Because time since SARS-CoV-2 infection strongly influences both antibody levels and T-cell responses, differences in infection timing between groups could contribute to the observed IFN-γ differences. If data on symptom history or timing of infection are available, adjustment or sensitivity analyses would strengthen the interpretation.

**ANSWER**: Dear reviewer, thank you for raising this point. We initially planned to include clinical data on previous infection including the timepoint into the analysis. However, it was very difficult to assess time of SARS-CoV-2 infection, as in fact only one individual reported a previous positive SARS-CoV-2 test result. This represents the limited testing possibilities at the time Gabon. Furthermore, from a clinical point, individuals could not provide history of SARS-CoV-2 specific symptoms. We therefore were not able to include any clinical data.

To address this we have added a sentence in the discussion.

**CHANGES** Line 446 – 451: Specific data on previous, laboratory confirmed SARS-CoV-2 infection was not available and clinical history did not allow to diagnose previous clinically apparent SARS-CoV-2 infection. Thus, it was not possible to assess any association between our findings and a history of previous COVID-19 disease or positive SARS-CoV-2 tests. Of course, this may limit the interpretation of the data as timepoint, and number of previous infections could have an impact on the immunological results.

**COMMENT**: Microfilaremic participants appear more likely to be male. Although sex was included as a covariate, it would be useful to confirm that the findings are robust in sex-stratified analyses.

**ANSWER:** Dear reviewer, indeed it is true that also in our study microfilaremic participants are predominantly male. This has been described in many Loa studies and represents the disease epidemiology in highly endemic populations. We did therefore explicitly test for sex as a confounder in our model and did not find evidence for different results according to sex (see table 2 and Supp. Info S5). We have now adapted the text for clarity.

**CHANGES:**

EG Line 259-260: These differences remained significant after adjustment for confounder age and sex (table 2).

307-308: This difference remained significant after adjustment for confounders age and sex (p= 0.031).

325-326: The observed difference between microfilaremic and occult remained significant after adjustment for confounders age and sex (p=0.012).

**COMMENT**: Given the relatively high prevalence of parasitic co-infections in the cohort, it would be useful to explore whether adjustment for co-infection status affects the observed associations.

**ANSWER:** Dear reviewer, thank you for raising this point. It has been raised throughout the revision, indicating that it needs to be better presented in the manuscript. However one needs to be aware that while we did screen for coinfections as well as possible allowed by study setting and funding- including Plasmodium parasitemia by thick blood smear, STH in stool and Schisto ova in urine, Mansonella pp in thick blood smears, etc - all these screening methods do have limited sensitivity for detection of active infection; so even a negative screening test may not allow to truly exclude active infection. Due to the limited sensitivity, we initially decided not to consider the screening results for co-infections in the analysis, but have now adapted the statistical analysis in the manuscript. Thus, we adjusted our models to the presence of co-infections. We have added a forest plots showing multivariate and univariate analysis of effects of coinfections on the outcome of INFy production (see Supp. Info. S5). For this analysis, we grouped the co-infections by expected immune response, leading to the following groups:

- Plasmodium spp.
- Schistosomiasis
- STH
- Mansonella spp.
- GI Protozoal infection

In this analysis we observed that our main results did not change after adjustment for uni and multivariate analysis.

We added sentences to address this in the methods, the results and the discussion and added the forest plots as Supp. Info. S5.

**CHANGES:** Line 228-233: Multiple linear regression models were fitted to assess group differences adjusted for age and sex as potential confounders. Further, models were adjusted to the presence of co-infections. For this analysis, co-infections were grouped by expected immune response into: Plasmodium spp., Schistosomiasis, Soil transmitted Helminths (STH) and intestinal protozoa. For an additional sensitivity analysis of IFN-γ assay results, multivariable models were adjusted for responder status (responder/non-responder).

Line 332-340: results: To further assess potential confounding by co-infections, we performed uni- and multivariable analyses including the major co-infections identified in the study population. Co-infections were grouped by pathogen type and their expected immune profile (Plasmodium parasitemia, Mansonella microfilaremia, STH ova in stool, urogenital Schistosomiasis, GI protozoan infection). Overall, the observed reduction in SARS-CoV-2-specific IFN- γ responses among microfilaremic individuals compared with those with occult loiasis remained robust across all different uni- and multivariable models examined (see Supp Info S5). Individuals with microscopically detectable Plasmodium spp. had generally lower INF- γ responses; however, the differences between microfilaremic and occult loiasis persisted.

Line 451-454: Next, despite thorough testing, further co-infections cannot be excluded and may have influenced immune responses. Additionally, in many tests used for detection of co-infection, sensitivity is limited. Therefore, some co-infections, even when tested for, may have not been detected.

**COMMENT**: Despite these limitations, the study addresses an interesting question regarding helminth-associated immune modulation. With additional analyses and a more cautious interpretation, the manuscript could make a useful contribution to understanding how chronic loiasis may influence immune responses to heterologous pathogens/antigens.

**ANSWER**: Dear reviewer, thank you very much for the review and feedback. We hope to have addressed all comments sufficiently and that you deem the manuscript appropriate for publication in the revised form.

**Reviewer #2:**

**COMMENT**: Summary: This is a very well written study with a robust experimental design. The main conclusion is an interesting finding of Loiasis being associated with reduced spike-specific T cell responses but not of spike-specific antibody levels. Key strengths of the study are the large number of individuals assessed, screening for other parasitic infecitons, and use of multivariable regression modeling to assess if the effect of Loiasis remains after controlling for possible confounders. No major limitations.

**ANSWER**: We thank the reviewer for their time, revision and helpful comments.

**COMMENT**: Major critique
A list of all the variables included in the multivariable regression analysis should be included in the methods (or depicted in a graph in the paper, see minor comment below).

**ANSWER:** Please find a more detailed response below.

**COMMENT**: Minor critique
Strongly recommend putting in a Forest plot that graphs the regression coefficients and the 95% confidence intervals obtained for each factor evaluated (both with univariate and multivariate modeling) to enable the reader to visualize the potential contribution of each factor towards IFNγ production to spike protein. Would comment in the results on the effects of some of the possible confounders (for example, did soil transmitted helminths or Schistosoma infection have as much of an effect as Loa loa).

**ANSWER:** Dear reviewer, thank you for raising this point. It has been raised throughout the revision, indicating that it needs to be better presented in the manuscript. However one needs to be aware that while we did screen for coinfections as well as possible allowed by study setting and funding- including Plasmodium parasitemia by thick blood smear, STH in stool and Schisto ova in urine, Mansonella pp in thick blood smears, etc - all these screening methods do have limited sensitivity for detection of active infection; so even a negative screening test may not allow to truly exclude active infection. Due to the limited sensitivity, we initially decided not to consider the screening results for co-infections in the analysis but have now adapted the statistical analysis in the manuscript. Thus, we adjusted our models to the presence of co-infections. We have added forest plots showing multivariate and univariate analysis of effects of coinfections on the outcome of INF-y production (see Supp. Info. S5). For this analysis, we grouped the co-infections by expected immune response, leading to the following groups:

- Plasmodium spp.
- Schistosomiasis
- STH
- Mansonella spp.
- GI Protozoal infection

In this analysis we observed that our main results did not change after adjustment for uni- and multivariate analyses.

We added sentences to address this in the methods, the results and the discussion and added the forest plots as supp. Info. S5.

**CHANGES:** Line 228-233: Multiple linear regression models were fitted to assess group differences adjusted for age and sex as potential confounders. Further, models were adjusted to the presence of co-infections. For this analysis, co-infections were grouped by expected immune response into: Plasmodium spp., Schistosomiasis, Soil transmitted Helminths (STH) and intestinal protozoa. For an additional sensitivity analysis of IFN-γ assay results, multivariable models were adjusted for responder status (responder/non-responder).

Line 332-340: results: To further assess potential confounding by co-infections, we performed uni- and multivariable analyses including the major co-infections identified in the study population. Co-infections were grouped by pathogen type and their expected immune profile (Plasmodium parasitemia, Mansonella microfilaremia, STH ova in stool, urogenital Schistosomiasis, GI protozoan infection). Overall, the observed reduction in SARS-CoV-2-specific IFN- γ responses among microfilaremic individuals compared with those with occult loiasis remained robust across all different uni- and multivariable models examined (see supp info S5). Individuals with microscopically detectable Plasmodium spp. had generally lower INF- γ responses; however, the differences between microfilaremic and occult loiasis persisted.

Line 451-454: Next, despite thorough testing, further co-infections cannot be excluded and may have influenced immune responses. Additionally, in many tests used for detection of co-infection, sensitivity is limited. Therefore, some co-infections, even when tested for, may have not been detected.

**Reviewer #3:**

**COMMENT**: The main goal of this study is to investigate how Loa loa infection influences the antibody and T cell responses to SARS-CoV2. It is a descriptive study, that doesn't include mechanist insights, and the immunological analysis are limited. However, the manuscript is well written, it has scientific value due to the scarcity of information regarding Loiasis and SARS-CoV2, and despite the limitations, they are well discussed.
However, among those limitations, one that can be better addressed in the analysis is the influence of co-infections in the immunological differences observed. Additional figures stratifying the groups regarding the presence of co-infection combined with microfilare status would strengthen the conclusion that the hyporesponsiveness is associated to the presence of microfilarae.

**ANSWER:** Dear reviewer, thank you for raising this point. It has been raised throughout the revision, indicating that it needs to be better presented in the manuscript. However one needs to be aware that while we did screen for coinfections as well as possible allowed by study setting and funding- including Plasmodium parasitemia by thick blood smear, STH in stool and Schisto ova in urine, Mansonella pp in thick blood smears, etc - all these screening methods do have limited sensitivity for detection of active infection; so even a negative screening test may not allow to truly exclude active infection. Due to the limited sensitivity, we initially decided not to consider the screening results for co-infections in the analysis but have now adapted the statistical analysis in the manuscript. Thus, we adjusted our models to the presence of co-infections. We have added forest plots showing multivariate and univariate analyses of effects of coinfections on the outcome of INF-y production (see Supp. Info. S5). For this analysis, we grouped the co-infections by expected immune response, leading to the following groups:

- Plasmodium spp.
- Schistosomiasis
- STH
- Mansonella spp.
- GI Protozoal infection

In this analysis we observed that our main results did not change after adjustment for uni and multivariate analysis.

We added sentences to address this in the methods, the results and the discussion and added the forest plots as supp. Info. S5.

**CHANGES:** Line 228-233: Multiple linear regression models were fitted to assess group differences adjusted for age and sex as potential confounders. Further, models were adjusted to the presence of co-infections. For this analysis, co-infections were grouped by expected immune response into: Plasmodium spp., Schistosomiasis, Soil transmitted Helminths (STH) and intestinal protozoa. For an additional sensitivity analysis of IFN-γ assay results, multivariable models were adjusted for responder status (responder/non-responder).

Line 332-340: results: To further assess potential confounding by co-infections, we performed uni- and multivariable analyses including the major co-infections identified in the study population. Co-infections were grouped by pathogen type and their expected immune profile (Plasmodium parasitemia, Mansonella microfilaremia, STH ova in stool, urogenital Schistosomiasis, GI protozoan infection). Overall, the observed reduction in SARS-CoV-2-specific IFN-γ responses among microfilaremic individuals compared with those with occult loiasis remained robust across all different uni- and multivariable models examined (see supp info S5). Individuals with microscopically detectable Plasmodium spp. had generally lower INF-γ responses; however, the differences between microfilaremic and occult loiasis persisted.

Line 451-454: Next, despite thorough testing, further co-infections cannot be excluded and may have influenced immune responses. Additionally, in many tests used for detection of co-infection, sensitivity is limited. Therefore, some co-infections, even when tested for, may have not been detected.

**Suggestions:**

**COMMENT**: Differences of eosinophils and lymphocytes were normalized based on sex and age. What happens with these parameters once you stratify the groups according to the presence of co-infections?

**ANSWER:** Thank you for this comment, we did assess for an influence of the co-infections on the eosinophils and leucocytes, however we did not find an influence on our results and the main findings remained robust. We have added another sentence in the results to address this:

**CHANGES** Line 260-263: Further, models were adjusted to the presence of co-infections and results remained significant (for absolute eosinophil counts p<0.001 for ML versus OL and p = 0.002 and ML versus NL, and for relative eosinophil counts p = 0.002 and p=0.003, respectively).

**COMMENT:** Additional analysis, for IgG and IFN, should be done stratifying the groups regarding the presence of co-infections. Each group should be divided into no-coinfection and any co-infection.

**ANSWER:** Dear reviewer, for us grouping all co-infections into one group would make less sense scientifically, as due to the differences in the pathogens we would expect very different immune responses (i.e. GI protozoa vs malaria etc), we therefore analyzed by group of co-infections as shown in the forest plot described above (Supp. Info S5).

**COMMENT**: Specifically for the IFN results it would be interesting to stratify the groups not necessarily regarding all the co-infections but separating the individuals that present co-infections that are known to induce type I immune responses.

**ANSWER**: We fully agree with the reviewer and think that the above mentioned and depicted groupings would make most sense from an immunological point of view.

-Besides the eosinophil, is there any correlation regarding the lymphocytes and the IFN?

**ANSWER**: We did not find a correlation between lymphocyte counts and IFNγ concentrations (r= 0.079 und p= 0.34).
