# Supplementary material for: Microfilaremic loiasis is associated with T cell hyporesponsiveness against SARS-CoV-2
Source: PLoS Negl Trop Dis. 2026 Oct 1;20(10):e0014104. doi: 10.1371/journal.pntd.0014104 (PMC13652322; doi:10.1371/journal.pntd.0014104)
Supplement: Peer review history

# Peer review: Decision Letter 0

**DOI:** 10.1371/journal.pntd.0014104.r001

**Authors:** Helton Santiago, Uriel Koziol

27 Mar 2026

Microfilaremic loiasis is associated with T cell hyporesponsiveness against SARS-CoV-2

PLOS Neglected Tropical Diseases

Dear Dr. Mackroth,

Thank you for submitting your manuscript to PLOS Neglected Tropical Diseases. After careful consideration, we feel that it has merit but does not fully meet PLOS Neglected Tropical Diseases's publication criteria as it currently stands. Therefore, we invite you to submit a revised version of the manuscript that addresses the points raised during the review process.

Please submit your revised manuscript within by May 26 2026 11:59PM. If you will need more time than this to complete your revisions, please reply to this message or contact the journal office at plosntds@plos.org. When you're ready to submit your revision, log on to https://www.editorialmanager.com/pntd/ and select the 'Submissions Needing Revision' folder to locate your manuscript file.

Please include the following items when submitting your revised manuscript:

* A letter that responds to each point raised by the editor and reviewer(s). You should upload this letter as a separate file labeled 'Response to Reviewers'. This file does not need to include responses to any formatting updates and technical items listed in the 'Journal Requirements' section below.

* A marked-up copy of your manuscript that highlights changes made to the original version. You should upload this as a separate file labeled 'Revised Manuscript with Track Changes'.

* An unmarked version of your revised paper without tracked changes. You should upload this as a separate file labeled 'Manuscript'.

If you would like to make changes to your financial disclosure, competing interests statement, or data availability statement, please make these updates within the submission form at the time of resubmission. Guidelines for resubmitting your figure files are available below the reviewer comments at the end of this letter.

We look forward to receiving your revised manuscript.

Kind regards,

Helton C. Santiago, M.D., Ph.D

Academic Editor

PLOS Neglected Tropical Diseases

Uriel Koziol

Section Editor

PLOS Neglected Tropical Diseases

Shaden Kamhawi

co-Editor-in-Chief

PLOS Neglected Tropical Diseases

orcid.org/0000-0003-4304-636XX

Paul Brindley

co-Editor-in-Chief

PLOS Neglected Tropical Diseases

orcid.org/0000-0003-1765-0002

**Additional Editor Comments:**

The manuscript was evaluated by three experts in the field who recognized the importance of the findings and suggested few further analysis or clarifications that the authors should address.

**Journal Requirements:**

1) Please upload all main figures as separate Figure files in .tif or .eps format. For more information about how to convert and format your figure files please see our guidelines:

https://journals.plos.org/plosntds/s/figures

2) When completing the data availability statement of the submission form, you indicated that you will make your data available on acceptance. We strongly recommend all authors decide on a data sharing plan before acceptance, as the process can be lengthy and hold up publication timelines. Please note that, though access restrictions are acceptable now, your entire data will need to be made freely accessible if your manuscript is accepted for publication. This policy applies to all data except where public deposition would breach compliance with the protocol approved by your research ethics board. If you are unable to adhere to our open data policy, please kindly revise your statement to explain your reasoning and we will seek the editor's input on an exemption. Please be assured that, once you have provided your new statement, the assessment of your exemption will not hold up the peer review process.

**Reviewers' Comments:**

Reviewer's Responses to Questions

**Key Review Criteria Required for Acceptance?**

As you describe the new analyses required for acceptance, please consider the following:

**Methods**

-Are the objectives of the study clearly articulated with a clear testable hypothesis stated?

-Is the study design appropriate to address the stated objectives?

-Is the population clearly described and appropriate for the hypothesis being tested?

-Is the sample size sufficient to ensure adequate power to address the hypothesis being tested?

-Were correct statistical analysis used to support conclusions?

-Are there concerns about ethical or regulatory requirements being met?

Reviewer #1: See general comments

Reviewer #2: Yes to all. Very well designed study.

Reviewer #3: (No Response)

**********

**Results**

-Does the analysis presented match the analysis plan?

-Are the results clearly and completely presented?

-Are the figures (Tables, Images) of sufficient quality for clarity?

Reviewer #1: See general comments

Reviewer #2: Yes. See notes below -- would likely benefit for inclusion of the results of univariate and multivariate modeling for all the possible confounders evaluated.

Reviewer #3: Suggestions:

- Differences of eosinophils and lymphocytes were normalized based on sex and age. What happens with these parameters once you stratify the groups according to the presence of co-infections?

- Additional analysis, for IgG and IFN, should be done stratifying the groups regarding the presence of co-infections. Each group should be divided into no-coinfection and any co-infection.

-Specifically for the IFN results it would be interesting to stratify the groups not necessarily regarding all the co-infections but separating the individuals that present co-infections that are known to induce type I immune responses.

-Besides the eosinophil, is there any correlation regarding the lymphocytes and the IFN?

**********

**Conclusions**

-Are the conclusions supported by the data presented?

-Are the limitations of analysis clearly described?

-Do the authors discuss how these data can be helpful to advance our understanding of the topic under study?

-Is public health relevance addressed?

Reviewer #1: See general comments

Reviewer #2: Yes.

Reviewer #3: (No Response)

**********

**Editorial and Data Presentation Modifications?**

Use this section for editorial suggestions as well as relatively minor modifications of existing data that would enhance clarity. If the only modifications needed are minor and/or editorial, you may wish to recommend “Minor Revision” or “Accept”.

Reviewer #1: See general comments

Reviewer #2: (No Response)

Reviewer #3: (No Response)

**********

**Summary and General Comments**

Use this section to provide overall comments, discuss strengths/weaknesses of the study, novelty, significance, general execution and scholarship. You may also include additional comments for the author, including concerns about dual publication, research ethics, or publication ethics. If requesting major revision, please articulate the new experiments that are needed.

Reviewer #1: This study addresses an important and understudied question regarding how chronic helminth infections such as Loa loa may influence immune responses to unrelated pathogens. Understanding such interactions is particularly relevant in endemic settings where helminth infections remain common and may influence responses to other infectious pathogens or vaccination.

The abstract states that the study was done in “Lambaréné and surrounding rural areas of Gabon” while the methods (lines 137-138) states “rural villages of the

138 Ngounié and Moyen-Ogooué provinces of Gabon”. Lambarene presumably includes urban populations sop this contradiction needs to be clarified.

RAPLOA was developed as a population-level mapping tool rather than a diagnostic instrument for individual classification. Because it relies on recall of of eyeworm migration, it may not reliably reflect current infection status. The resulting “occult loiasis” category likely will include biologically heterogeneous individuals, including those whose immune systems control microfilaremia more effectively and who mount stronger anti-parasite immune responses. Such individuals might also mount stronger IFN-γ responses to unrelated antigens, including SARS-CoV-2. Sensitivity analyses could help address this issue, including (i) comparing microfilaremia versus no microfilaremia independent of RAPLOA stratification and (ii) examining correlations between microfilarial load and IFN-γ responses among microfilaremic individuals.

Could the authors provide a clearer explanation for the following: The relative validation criteria for the IFN-γ release assay led to some Quan-T-Cell-test-negative individuals having a higher measured IFN-γ concentration than some positive individuals”.

A potentially important issue relates to the exclusion of IFN-g-‘negative’ individuals from analysis. Because IFN-γ responsiveness is the outcome of interest, restricting the analysis to individuals with detectable responses means selecting participants based on the outcome itself, which may introduce bias. An alternative approach could be to analyse all participants and model either responder status or IFN-γ concentrations including zero values or to model response probability (responder vs. non-responders).

Details should be provided of the laboratory detection of STH/Schistosoma in stool and urine samples

Because time since SARS-CoV-2 infection strongly influences both antibody levels and T-cell responses, differences in infection timing between groups could contribute to the observed IFN-γ differences. If data on symptom history or timing of infection are available, adjustment or sensitivity analyses would strengthen the interpretation.

Microfilaremic participants appear more likely to be male. Although sex was included as a covariate, it would be useful to confirm that the findings are robust in sex-stratified analyses.

Given the relatively high prevalence of parasitic co-infections in the cohort, it would be useful to explore whether adjustment for co-infection status affects the observed associations.

Despite these limitations, the study addresses an interesting question regarding helminth-associated immune modulation. With additional analyses and a more cautious interpretation, the manuscript could make a useful contribution to understanding how chronic loiasis may influence immune responses to heterologous pathogens/antigens.

Reviewer #2: Summary: This is a very well written study with a robust experimental design. The main conclusion is an interesting finding of Loiasis being associated with reduced spike-specific T cell responses but not of spike-specific antibody levels. Key strengths of the study are the large number of individuals assessed, screening for other parasitic infecitons, and use of multivariable regression modeling to assess if the effect of Loiasis remains after controlling for possible confounders. No major limitations.

Major critique

A list of all the variables included in the multivariable regression analysis should be included in the methods (or depicted in a graph in the paper, see minor comment below).

Minor critique

Strongly recommend putting in a Forest plot that graphs the regression coefficients and the 95% confidence intervals obtained for each factor evaluated (both with univariate and multivariate modeling) to enable the reader to visualize the potential contribution of each factor towards IFNg production to spike protein. Would comment in the results on the effects of some of the possible confounders (for example, did soil transmitted helminths or Schistosoma infection have as much of an effect as Loa loa).

Reviewer #3: The main goal of this study is to investigate how Loa loa infection influences the antibody and T cell responses to SARS-CoV2. It is a descriptive study, that doesn't include mechanist insights, and the immunological analysis are limited. However, the manuscript is well written, it has scientific value due to the scarcity of information regarding Loiasis and SARS-CoV2, and despite the limitations, they are well discussed.

However, among those limitations, one that can be better addressed in the analysis is the influence of co-infections in the immunological differences observed. Additional figures stratifying the groups regarding the presence of co-infection combined with microfilare status would strengthen the conclusion that the hyporesponsiveness is associated to the presence of microfilarae.

**********

PLOS authors have the option to publish the peer review history of their article (what does this mean?). If published, this will include your full peer review and any attached files.

If you choose “no”, your identity will remain anonymous but your review may still be made public.

**Do you want your identity to be public for this peer review?**  For information about this choice, including consent withdrawal, please see our Privacy Policy.

Reviewer #1: No

Reviewer #2: No

Reviewer #3: No

[NOTE: If reviewer comments were submitted as an attachment file, they will be attached to this email and accessible via the submission site. Please log into your account, locate the manuscript record, and check for the action link "View Attachments". If this link does not appear, there are no attachment files.]

**Figure resubmission:**

**Reproducibility:**

To enhance the reproducibility of your results, we recommend that authors of applicable studies deposit laboratory protocols in protocols.io, where a protocol can be assigned its own identifier (DOI) such that it can be cited independently in the future. Additionally, PLOS ONE offers an option to publish peer-reviewed clinical study protocols. Read more information on sharing protocols at https://plos.org/protocols?utm_medium=editorial-email&utm_source=authorletters&utm_campaign=protocols

# Author comment: Author response to Decision Letter 1

**DOI:** 10.1371/journal.pntd.0014104.r002

13 Jun 2026

Attachment Submitted filename: Mackroth_Microfil.loiasis_and_SarsCoV2_response_to_reviewer.docx

# Peer review: Decision Letter 1

**DOI:** 10.1371/journal.pntd.0014104.r003

**Authors:** Uriel Koziol

17 Sep 2026

Dear Dr Mackroth,

We are pleased to inform you that your manuscript 'Microfilaremic loiasis is associated with T cell hyporesponsiveness against SARS-CoV-2' has been provisionally accepted for publication in PLOS Neglected Tropical Diseases.

Before your manuscript can be formally accepted you will need to complete some formatting changes, which you will receive in a follow up email. A member of our team will be in touch with a set of requests.

Please note that your manuscript will not be scheduled for publication until you have made the required changes, so a swift response is appreciated.

IMPORTANT: The editorial review process is now complete. PLOS will only permit corrections to spelling, formatting or significant scientific errors from this point onwards. Requests for major changes, or any which affect the scientific understanding of your work, will cause delays to the publication date of your manuscript.

Should you, your institution's press office or the journal office choose to press release your paper, you will automatically be opted out of early publication. We ask that you notify us now if you or your institution is planning to press release the article. All press must be co-ordinated with PLOS.

Thank you again for supporting Open Access publishing; we are looking forward to publishing your work in PLOS Neglected Tropical Diseases.

Best regards,

Uriel Koziol

Section Editor

PLOS Neglected Tropical Diseases

Uriel Koziol

Section Editor

PLOS Neglected Tropical Diseases

Shaden Kamhawi

co-Editor-in-Chief

PLOS Neglected Tropical Diseases

orcid.org/0000-0003-4304-636XX

Paul Brindley

co-Editor-in-Chief

PLOS Neglected Tropical Diseases

orcid.org/0000-0003-1765-0002

***********************************************************

Reviewer's Responses to Questions

**Key Review Criteria Required for Acceptance?**

As you describe the new analyses required for acceptance, please consider the following:

**Methods**

-Are the objectives of the study clearly articulated with a clear testable hypothesis stated?

-Is the study design appropriate to address the stated objectives?

-Is the population clearly described and appropriate for the hypothesis being tested?

-Is the sample size sufficient to ensure adequate power to address the hypothesis being tested?

-Were correct statistical analysis used to support conclusions?

-Are there concerns about ethical or regulatory requirements being met?

Reviewer #1: (No Response)

Reviewer #2: Yes to all.

Reviewer #3: (No Response)

**********

**Results**

-Does the analysis presented match the analysis plan?

-Are the results clearly and completely presented?

-Are the figures (Tables, Images) of sufficient quality for clarity?

Reviewer #1: (No Response)

Reviewer #2: Yes to all.

Reviewer #3: (No Response)

**********

**Conclusions**

-Are the conclusions supported by the data presented?

-Are the limitations of analysis clearly described?

-Do the authors discuss how these data can be helpful to advance our understanding of the topic under study?

-Is public health relevance addressed?

Reviewer #1: (No Response)

Reviewer #2: Yes to all.

Reviewer #3: (No Response)

**********

**Editorial and Data Presentation Modifications?**

Use this section for editorial suggestions as well as relatively minor modifications of existing data that would enhance clarity. If the only modifications needed are minor and/or editorial, you may wish to recommend “Minor Revision” or “Accept”.

Reviewer #1: (No Response)

Reviewer #2: n/a

Would accept this revised version.

Reviewer #3: (No Response)

**********

**Summary and General Comments**

Use this section to provide overall comments, discuss strengths/weaknesses of the study, novelty, significance, general execution and scholarship. You may also include additional comments for the author, including concerns about dual publication, research ethics, or publication ethics. If requesting major revision, please articulate the new experiments that are needed.

Reviewer #1: There still remains the issue of RAPLOA-positive group being heterogeneous. The authors should acknowledge this more explicitly in the Discussion and avoid implying that the occult group represents a biologically homogeneous clinical entity.

The issue of residual confounding should be stated more explicitly in limitations.

Reviewer #2: The authors have done a good job responding to the comments.

Reviewer #3: All my concerns were answered.

**********

PLOS authors have the option to publish the peer review history of their article (what does this mean?). If published, this will include your full peer review and any attached files.

If you choose “no”, your identity will remain anonymous but your review may still be made public.

**Do you want your identity to be public for this peer review?**  For information about this choice, including consent withdrawal, please see our Privacy Policy.

Reviewer #1: No

Reviewer #2: No

Reviewer #3: No

# Editor report: Acceptance letter

**DOI:** 10.1371/journal.pntd.0014104.r004

**Authors:** Uriel Koziol

Dear Dr Mackroth,

We are delighted to inform you that your manuscript, "Microfilaremic loiasis is associated with T cell hyporesponsiveness against SARS-CoV-2," has been formally accepted for publication in PLOS Neglected Tropical Diseases.

We have now passed your article onto the PLOS Production Department who will complete the rest of the publication process. All authors will receive a confirmation email upon publication.

The corresponding author will soon be receiving a typeset proof for review, to ensure errors have not been introduced during production. Please review the PDF proof of your manuscript carefully, as this is the last chance to correct any scientific or type-setting errors. Please note that major changes, or those which affect the scientific understanding of the work, will likely cause delays to the publication date of your manuscript. Note: Proofs for Front Matter articles (Editorial, Viewpoint, Symposium, Review, etc...) are generated on a different schedule and may not be made available as quickly.

Soon after your final files are uploaded, the early version of your manuscript will be published online unless you opted out of this process. The date of the early version will be your article's publication date. The final article will be published to the same URL, and all versions of the paper will be accessible to readers.

For Research Articles, you will receive an invoice from PLOS for your publication fee after your manuscript has reached the completed accept phase. If you receive an email requesting payment before acceptance or for any other service, this may be a phishing scheme. Learn how to identify phishing emails and protect your accounts at https://explore.plos.org/phishing.

Thank you again for supporting open-access publishing; we are looking forward to publishing your work in PLOS Neglected Tropical Diseases.

Best regards,

Shaden Kamhawi

co-Editor-in-Chief

PLOS Neglected Tropical Diseases

Paul Brindley

co-Editor-in-Chief

PLOS Neglected Tropical Diseases
